# Association between depression and fruit and vegetable consumption among adults in South Asia

**DOI:** 10.1186/s12888-017-1198-1

**Published:** 2017-01-14

**Authors:** Ghose Bishwajit, Daniel Peter O’Leary, Sharmistha Ghosh, Yaya Sanni, Tang Shangfeng, Feng Zhanchun

**Affiliations:** 1 Institute of Nutrition and Food Science, University of Dhaka, Dhaka, 1000 Bangladesh; 2 School of Psychology, Bangor University, Bangor, Wales, UK; 3 Department of Sociology, University of Dhaka, Dhaka, 1000 Bangladesh; 4 School of International Development and Global Studies, University of Ottawa, Ottawa, Canada; 5 School of Medicine and Health Management, Tongji Medical College, Huazhong University of Science and Technology, Wuhan, China

**Keywords:** Fruit and vegetable consumption, Depression, South Asia, World health survey, Self-reported depression

## Abstract

**Background:**

In recent years there has been a growing research interest regarding the impact of dietary behaviour on mental health outcomes. The present study aimed to investigate the association between fruit and vegetable (F&V) consumption and depression in three south Asian countries- Bangladesh, India and Nepal.

**Methods:**

Cross-sectional data were obtained from World Health Survey of WHO conducted during 2002–04. In total 14,133 adult subjects (Bangladesh 3262, India 7594, Nepal 3277) aged 18 years and above were included in the study. Outcome variables were Self-Reported Depression (SRD) during last 30 days and 12 months. Multivariable regression methods were used to explore the association between F&V consumption and depression.

**Results:**

Prevalence of Self-Reported Depression during past 12 months were respectively 39%, 17.7%, and 49.9% for Bangladesh, India and Nepal. In India, those who consumed less than five servings of vegetables were respectively 41% [AOR = 1.41; 95%CI = 0.60-3.33] and 57% [AOR = 1.57; 95%CI = 0.93-2.64] more likely to report severe-extreme and mild-moderate depression during past 30 days compared to those who consumed five servings a day. Regarding fruit consumption, compared to those who consumed five servings a day, the odds of severe-extreme and mild-moderate SRD were respectively 3.5 times [AOR = 3.48; 95%CI = 1.216-10.01] and 45% [AOR = 1.44; 95%CI = 0.89-2.32] higher in Bangladesh, and 2.9 times [AOR = 2.92; 95%CI = 1.12-7.64] and 42% higher [AOR = 1.41; 95%CI = 0.89-2.24] in Nepal compared to those who consumed less than five servings a day during last 30 days.

**Conclusion:**

Daily intake of less than five servings of F&V was associated with higher odds of depression. Nutrition programs aimed at promoting F&V consumption might prove beneficial to reduce the prevalence of depression in south Asian population. Further studies are required to understand the factors limiting the adequate consumption of F&V.

## Background

Depression represents a major public health concern worldwide and it is often referred as the common cold in the field of psychiatry. This analogy may provide a good understanding of the frequency of occurrence, however its significance goes far deeper with repercussions on academic and professional performance, Quality of Life (QoL), familial and Social Well Being (SWB). Global Burden of Disease (GBD) 2010 Study reported a global prevalence of Major Depressive Disorder (MDD) of 4.7% (4.4–5.0%) with an incidence of 3.0% (2.4–3.8%) [1]. Moussavi et al. reported a lifetime prevalence of depression of 15 to 20% globally [2]. Another Global Burden of Disease (GBD) 2010 study reported that MDD ranked 11^th^ among the leading causes of Disability Adjusted Life Years (DALYs) worldwide in 2010, a 37% increase since 1990 (15^th^ in 1990) [3]. Worldwide, about 25% of individuals develop one or more mental or behavioral disorders during their lifetime [4]. In 2002, depression was the third leading cause of disease burden (equivalent to 4.3% of all DALYs), and also the leading cause of disability responsible for 13.4% of Years Lived with Disability (YLDs) in women and 8.3% in men [5]. According to the World Health Organization, depression is projected to be a leading cause of disability worldwide by 2020, second only to ischemic health disease [6].

There is a growing volume of research dedicated to investigating the epidemiology and rising prevalence of depression, the risk factors, and devising preventive and intervention measures. Some have attributed the increasing prevalence to the changing lifestyle brought by modernity and to the depressiogenic/stressogenic environment it has brought along e.g. dietary changes, urbanization, social inequality and isolation, loneliness, sedentary lifestyle, sleep-deprivation [7–9]. Certain lifestyle related issues and adoption of unhealthy behaviours function as contributing factors to poor physical health outcomes and give rise to higher incidence of psychological disorders [7–10]. Pharmacological treatments of depressive disorders have experienced remarkable progress over the course of past 4–5 decades and constitutes to be the main therapeutic approach for depression. However, non-pharmacological management (e.g. dietary behaviour, physical activity) of psychological disorders are also gaining increasing attention. For instance, there has been a renewed interest in the potential role of dietary management such as fruit and vegetables consumption in preventing Non-communicable Chronic Disease (NCDs) including mental illnesses [11, 12]. According to some estimates, inadequate fruit consumption is the most prominent dietary risk factor for global disease burden and responsible for about 4.9 million (95% CI 3.8–5.9) deaths and 4.2% (95% CI 3.3–5.0) of global DALYs [13].

Fruits and vegetables are regarded as essential components of a healthy diet for their low energy content and rich sources of micronutrients, fiber, and other large number of bioactive compounds with potential effect on brain and overall health [14]. One widely accepted mechanism for higher fruits and vegetables consumption on better mental health is that antioxidants defend against the negative effects of oxidative stress, which is associated with depression [15, 16]. Moreover, antioxidants are shown to have beneficial effects on inflammatory markers which are associated with elevated levels of depression [17]. Regular consumption of fruits and vegetables can help body fight against the causative agents and cope up with depressive syndromes. Dietary guidelines by WHO/FAO recommends a minimum of 5 servings (400 g) of F&V/day that provides a reasonable amount of micronutrients which may contribute to favorable cardiometabolic outcomes [18]. However, in many Low and Middle-Income Counties (LMICs) the level of F&V intake is far lower than this level. In South Asia for instance, F&V intake among adults in India and Pakistan was reported at about 100 g per capita per day or less, compared to 300 g in Europe and the USA [19]. Country level data on F&V consumption are not available, however different sources suggest that average number of vegetable servings on the days when vegetable was consumed were of 3–3.4 servings in Matlab, and 1.3-1.5 servings in Vadu, India [19]. A multi-country study reported 74% lower than recommended level of F&V consumption among adult population in India [20]. Though several researches have provided evidence on the role of F&V consumption in the prevention of chronic diseases on South Asian population, there is no study so far conducted in the context of psychological disorders. With an aim to address this gap, we conducted this study exploring the association between the frequency of F&V consumption and Self-Reported Depression. It should be noted that data on dietary pattern and mental illness are very limited in this region. We utilised datasets from the World Health Survey (2002–04) which is the first to provide country representative data on these indicators in South Asia.

## Methods

### The survey

This study is based on data extracted from World Health Survey of WHO conducted between 2002 and 2004 which are available from WHO upon request. The program is operational in 70 countries including four south Asian nations namely Bangladesh, India, Nepal and Sri Lanka. Objectives of the WHO funded survey were to provide reliable, nationally comparable data on a wide range of health and socioeconomic indicators that are necessary for monitoring performance and responsiveness of health systems progress towards public health related goals [21]. The target population were randomly selected male and female adults aged 18 years or over residing in non-institutional settings (e.g. excluding military reservations, or other non-household living arrangements). For those who were in a health institution (e.g. hospital, hospice, nursing home, home for the aged, etc.) at the time of household visit, interview was conducted either in the institution or upon their return to their household if within a period of two weeks from the first visit to the household.

The interviews were done face-to-face in the local language using pencil and paper questionnaires. Each interview lasted for approximately sixty minutes depending on the comprehension and literacy level of the respondent. Interviews were conducted by qualified personnel familiar with the local culture, customs and the language. Multistage cluster sampling method was employed to include eligible individuals and the number of individuals selected were 5924 for Bangladesh (response rate 94%), 9977 for India (response rate 97%), 8818 for Nepal (response rate 98%), 6759 for Sri Lanka (response rate 99%). Further details regarding the survey methods are available elsewhere [22].

#### Outcome

Self-Reported Depression (SRD) status during last 30 days and 12 months were the outcome variable in this study.

Respondents were asked- During the last 12 months, have you had a period lasting several days when you felt sad, empty or depressed. Self-reported response categories to these question was- 1. Yes, 2. No.

For short term depression, the question was- Overall in the last 30 days, how much of a problem did you have with feeling sad, low or depressed? Possible answers to this question were: 1. None 2. Mild 3. Moderate 4. Severe, and 5. Extreme. For regression analysis, the categories were collapsed into three: Not depressed, Mild-Moderate Depression, and Severe-Extreme Depression.

The explanatory variable of primary interest was fruit and vegetable consumption. Respondents were asked: How many servings of fruit do you eat on a typical day?

Answer ranged from 0 to 14 servings a day. As per WHO/FAO recommendation, the cut-off of at least five servings of F&V a day was used, and the following categorisation was used: <5 servings a day/5 servings a day every day/>5 servings a day.

Based on literature review and availability on the datasets, the other explanatory variables included in the study were- Age: 18-29/30-39/40-49/50-59/60+ years; Sex: Female/Male; Currently married: No/Yes; Educational attainment: Nil/Less than primary school/Primary complete/Secondary complete/High school/equivalent complete/Pre-university/University; Employment status: Government employee/Private employee/Employer/Unemployed; Smoking habit: Daily/Yes but not daily/Non-smoker; Ever drank alcohol: Yes/No, Satisfaction with health: Very dissatisfied/Dissatisfied/Neither Satisfied nor dissatisfied/Satisfied/Very Satisfied.

### Statistical analysis

Datasets were checked for missing values, outliers and were weighted to ensure the results are representative of the population. Variables were also categorised before analysis. Sample characteristics were analysed through simple descriptive statistics e.g. frequencies and percentages. Cross tabulation was performed to measure the distribution of the sociodemographic variables across the outcome variable and crude prevalence of depression. Significance of group differences (depressed Vs not depressed) for the explanatory variables were tested by chi-square tests and was presented as *p*-values. Final step was regression analysis that assessed the adjusted associations between depression and F&V consumption. Only the variables that had a *p*-value below 0.025 in the cross-tabs were selected for the regression analysis [23]. Three separate regression models were run for each country. The outcomes of the regression analysis were reported in terms of adjusted odds ratios (AOR) and corresponding 95% confidence intervals. Analyses were performed with SPSS version 21 and Stata version 12.

## Results

Basic socioeconomic and demographic characteristics of the study population are presented in Table 1. Among all three countries, Nepal had the highest mean age (42.65 years, SD 16.58) and India had lowest (39.11, SD 15.32). Majority of the sample population for all the countries were aged below 30 years and were female. Rate of being currently married was respectively 57.2%, 76.8% and 80.8% for Bangladesh, India and Nepal. Literacy rate was highest for highest for India (41.4%) and lowest for Nepal (31.1%). Unemployment rate was respectively 54.9%, 45.7% and 33.1% for Bangladesh, India and Nepal, and majority of the participants were self-employed. Tobacco smoking was more prevalent than alcohol drinking in all three countries- Bangladesh (45.8 Vs 6.5%), India (34.3 Vs 10.5%), Nepal (45.5 Vs 36.1%). Rate of satisfaction (satisfied and very satisfied) with health was respectively 42.7%, 56.6%, 41.9% for Bangladesh, India and Nepal. Rate of adequate amount of fruit and vegetable consumption was very low for all three countries. Percentage of sample population consuming 5/5+ servings of fruits was respectively 5.1% for both Bangladesh, India and 8.6% for Nepal, and that for vegetable consumption was respectively 13.8%, 5.6%, and 2.7% for Bangladesh, India and Nepal.

**Table 1:** Basic sociodemographic characteristics of the sample population

Variables	Bangladesh	India	Nepal
Age Mean(SD)	39.66 (15.31)	39.11 (15.32)	42.65 (16.58)
18–29	28.6	30.7	24.7
30–39	26.4	25.2	23.6
40–49	20.6	18.0	19.0
50–59	11.4	12.3	12.5
60+	13.0	13.7	20.2
Sex			
Female	57.2	52.2	63.1
Male	42.8	47.8	36.9
Currently married			
Yes	57.2	76.8	80.8
No	42.8	23.2	19.2
Educational attainment			
Nil	44.2	39.6	69.3
Primary	43.1	27.0	21.5
Secondary	9.7	23.0	8.5
Pre-university/University	3.0	10.3	0.6
Job			
Govt. employee	2.7	4.7	2.3
Private employee	5.5	10.4	1.5
Employer	36.9	39.3	63.0
Not working for payment	54.9	45.7	33.1
Smoking habit			
Daily	41.0	31.2	39.2
Yes. not daily	4.8	3.0	6.3
Non-smoker	54.2	65.7	54.5
Alcohol			
Yes	6.5	10.5	36.1
No	93.5	89.5	63.9
Satisfaction with health			
Very dissatisfied	7.9	5.1	6.8
Dissatisfied	18.1	14.9	23.3
Neither	31.3	21.5	28.0
Satisfied	35.5	48.8	38.5
Very Satisfied	7.2	9.8	3.4
Fruit consumption			
<5	94.9	94.9	91.4
5	2.5	2.2	6.5
5+	2.6	2.9	1.1
Vegetable consumption			
<5	86.2	94.4	97.3
5	4.2	1.4	1.5
5+	9.6	4.3	1.2

### Prevalence of self-reported depression and its association with the explanatory variables

Table 2 indicates that prevalence of Self-Reported Depression (SRD) was respectively 39%, 17.7%, and 49.9% for Bangladesh, India and Nepal. SRD tended to be more prevalent among the younger age groups, female, currently unmarried, having no formal education, having no employment, smoking tobacco and drinking alcohol. Those who reported being very satisfied with health and consuming 5 or 5+ servings of fruits and vegetables were less likely to report suffering from depression.

**Table 2:** Percentage of population reporting depression during past 12 months, World Health Survey, 2002–03

Variables	Bangladesh (39)	India (17.7)	Nepal (49.5)
Age Mean			
18–29	25.2	17.0	21.6
30–39	11.4	20.9	21.4
40–49	19.3	20.0	18.2
50–59	18.3	19.5	14.0
60+			
p	<0.001	<0.001	<0.001
Sex			
Female	66.2	56.3	66.2
Male	33.8	43.7	33.8
	<0.001	<0.001	<0.001
Currently married			
Yes	25.7	21.5	21.0
No	74.3	78.5	79.0
p	<0.001	0.054	0.005
Educational attainment			
Nil	48.9	49.2	75.2
Primary	41.4	29.8	18.4
Secondary	7.9	14.6	6.0
Pre-university/University	1.9	6.4	0.5
p	<0.001	<0.001	<0.001
Job			
Govt. employee	1.8	2.6	1.7
Private employee	3.9	8.9	1.1
Employer	31.1	38.2	37.6
Not working for payment	63.1	50.3	59.5
p	<0.001	<0.001	<0.001
Smoking habit			
Daily	56.4	58.0	53.0
Yes. not daily	4.8	4.2	6.5
Non-smoker	38.8	37.7	40.5
p	0.118	<0.001	0.211
Alcohol			
No	5.8	12.9	36.2
Yes	94.2	87.1	63.8
p	0.117	0.001	0.451
Satisfaction with health			
Very dissatisfied	11.4	9.5	8.7
Dissatisfied	23.3	27.1	27.3
Neither	33.6	27.1	27.3
Satisfied	25.4	31.5	33.4
Very Satisfied	6.4	4.7	3.3
	<0.001	<0.001	<0.001
Fruit consumption			
<5	96.3	94.5	93.2
5	2.2	2.2	6.7
5+	1.5	3.3	0.1
p	0.004	<0.001	0.177
Vegetable consumption			
<5	86.9	95.7	97.3
5	3.4	1.6	1.5
5+	9.7	2.7	1.2
p	0.156	0.005	0.139

Prevalence of SRD in past 30 days was shown in table 3. Nepal had the highest prevalence of severe to extreme SRD (17.2%) and India had the lowest (11.9%). Mild to moderate SRD was most prevalent in Bangladesh (44.7%) followed by Nepal (36.4%) and India (33.3%). Similar to SRD during past 12 months, that during past 30 days were more prevalent among those were elderly, female, currently unmarried, had no formal education and employment, smoked tobacco, drank alcohol and less prevalent among those who reported satisfaction with health and consuming 5/5+ servings of fruits and vegetables every day.

**Table 3:** Percentage of population reporting depression in past 30 days, World Health Survey, 2002–03

Variables	Bangladesh		India		Nepal	
	Severe-extreme	Mild-moderate	Severe-extreme	Mild-moderate	Severe-extreme	Mild-moderate
	14.7	44.7	11.9	33.3	17.2	36.4
Age						
18–29	15.1	12.7	17.2	18.4	10.4	18.2
30–39	18.2	12.1	20.1	15.2	10.2	21.9
40–49	17.2	22.6	19.3	18.4	18.4	20.9
50–59	17.4	25.8	13.7	23.5	27.6	15.5
60+	32.2	26.8	29.7	24.5	33.4	23.6
p	<0.001	<0.001	<0.001	<0.001	<0.001	<0.001
Sex						
Female	71.8	61.0	64.4	56.2	60.0	65.1
Male	28.2	39.0	35.6	43.8	40.0	34.9
p	<0.001	<0.001	<0.001	<0.001	0.002 < 0.001	
Currently married						
Yes	33.5	20.5	26.4	21.2	15.5	20.5
No	66.5	79.5	73.6	78.8	84.5	79.5
p	<0.001	<0.001	<0.001 0.003		<0.001	<0.001
Educational attainment						
Nil	54.6	46.0	55.7	46.3	61.2	75.0
Primary	37.7	42.6	28.0	29.1	26.1	18.9
Secondary	6.1	8.9	12.1	18.6	11.9	5.9
Pre-university/University	1.7	2.5	4.2	6.0	0.9	0.2
p	<0.001	<0.001	<0.001	<0.001	<0.001	<0.001
Employment status						
Govt. employee	1.5	2.9	1.9	3.5	2.7	2.3
Private employee	2.5	4.7	6.3	9.1	1.8	1.5
Employer	23.4	35.4	35.9	39.1	68.9	62.6
Not working for payment	72.6	57.0	55.9	48.3	26.6	33.5
p	<0.001	<0.001	<0.001	<0.001	<0.001	<0.001
Smoking habit						
Daily	49.0	52.9	60.4	62.8	57.3	51.2
Yes. not daily	5.0	4.9	2.7	3.7	6.1	6.4
Non-smoker	46.0	42.2	36.9	33.5	36.6	42.4
p	0.017 < 0.001		<0.001	<0.001	<0.001 0.032	
Alcohol						
No	10.3	5.5	9.9	11.3	32.7	40.3
Yes	89.7	94.5	90.1	88.7	67.3	59.7
p	<0.001	<0.001	0.294	<0.001	<0.001	<0.001
Satisfaction with health						
Very dissatisfied	16.9	6.4	12.7	4.8	4.9	6.7
Dissatisfied	28.2	21.2	32.6	20.5	17.5	26.0
Neither	27.8	36.2	24.4	29.1	25.4	30.7
Satisfied	22.8	30.6	26.1	40.8	48.5	33.9
Very Satisfied	4.2	5.6	4.2	4.8	3.7	2.6
p	<0.001 < 0.001		<0.001	<0.001	<0.001	<0.001
Fruit consumption						
<5	94.8	95.5	95.1	95.6	92.8	89.8
5	3.6	2.2	2.2	2.1	5.7	7.7
5+	1.7	2.3	2.7	2.3	1.6	2.5
P	0.068	<0.001	<0.001 0.169		<0.001 0.001	
Vegetable consumption						
<5	88.9	85.9	95.2	94.3	96.2	97.4
5	3.3	4.0	1.2	1.7	2.2	1.2
5+	7.7	10.1	3.6	4.0	1.6	1.4
P	<0.001 0.135		<0.001 0.03		<0.001 0.15	

### Association between fruit and vegetable consumption and SRD

Results of multivariable regression analysis for the association between frequency of fruit and vegetable consumption during past 12 months and 30 days with SRD were shown in Tables 4 and 5. Results indicate that compared to those who consumed five servings of vegetables per day, those who consumed less than five servings had 51% [AOR = 1.51; 95%CI = 0.82-2.76] higher odds of reporting depression during past 12 months in India. In Bangladesh, consuming more than five servings of vegetables decreased the odds of SDR by 32% [AOR = 0.67; 95%CI = 0.44-1.03]. Compared to those who consumed five servings of fruits per day, the odds of SDR were respectively 86% and 3.1 times higher in Bangladesh and India among those who consumed less than five servings.

**Table 4:** Association between frequency of fruit and vegetable consumption and Self-Reported Depression during past 12 months in Bangladesh, India and Nepal. World Health Survey, 2002–03

	Bangladesh	India	Nepal
Vegetable consumption	AOR (95%CI)	AOR (95%CI)	AOR (95%CI)
5	-	-	-
<5	0.97 (0.48-2.01)	1.51 (0.82-2.76)	0.98 (0.17-3.6)
5+	0.67 (0.44-1.03)	1.08 (0.73-1.90)	0.99 (0.57-1.72)
Fruit consumption			
5	-	-	-
<5	1.85 (0.93-3.69)	3.10 (1.57-6.10)	0.89 (0.47-1.61)
5+	0.81 (0.51-1.30)	1.10 (0.741-1.65)	1.06 (0.79-1.42)

N.B. *AOR* Adjusted odds ratio, *CI* Confidence Interval. Adjusted for variable with a *p*-value less than 0.25 in the chi-square tests

**Table 5:** Association between frequency of fruit and vegetable consumption during past 30 days and Self-Reported Depression in Bangladesh, India and Nepal. World Health Survey, 2002–03

Variables	Bangladesh		India		Nepal	
	Severe-extreme	Mild-moderate	Severe-extreme	Mild-moderate	Severe-extreme	Mild- moderate
Vegetable consumption	AOR (95%CI)	AOR (95%CI)	AOR (95%CI)	AOR (95%CI)	AOR (95%CI)	AOR (95%CI)
5						
<5	1.06 (0.7-1.61)	0.96 (0.55-1.34)	1.414 (0.60-3.33)	1.57 (0.93-2.64)	0.89 (0.44-1.66)	0.93 (0.72-1.19)
5+	0.967 (0.47-1.96)	0.934 (0.72-1.21)	1.018 (0.81-2.13)	1.06 (0.78-1.43)	1.183 (0.87-1.88)	0.898 (0.50-1.17)
Fruit consumption						
5						
<5	3.48 (1.21-10.01)	1.44 (0.89-2.32)	0.89 (0.34-1.04)	1.08 (0.74-1.57)	2.929 (1.12-7.64)	1.41 (0.89-2.24)
5+	1.127 (0.96-5.14)	1.172 (0.68-2.74)	0.948 (0.31-2.34)	1.001 (0.61-1.63)	1.076 (0.87-4.02)	1.11 (0.66-2.53)

N.B. *AOR* Adjusted odds ratio, *CI* Confidence Interval. Adjusted for variable with a *p*-value less than 0.25 in the chi-square tests

In India, those who consumed less than five servings of vegetables were respectively 41% [AOR = 1.41; 95%CI = 0.60-3.33] and 57% [AOR = 1.57; 95%CI = 0.93-2.64] more likely to report severe-extreme and mild-moderate depression during past 30 days compared to those who consumed five servings a day.

Regarding fruit consumption, compared to those who consumed five servings a day, the odds of severe-extreme and mild-moderate SDR were respectively 3.5 times [AOR = 3.48; 95%CI = 1.21-10.01] and 45% [AOR = 1.44; 95%CI = 0.89-2.32] higher in Bangladesh, and 2.9 times [AOR = 2.92; 95%CI = 1.12-7.64] and 42% higher [AOR = 1.41; 95%CI = 0.89-2.24] in Nepal among those who consumed less than five servings a day during last 30 days.

## Discussion

Findings indicate that Nepal had the highest rate of SRD both in the past 12 months and 30 days followed by Bangladesh and India. However, prevalence of mild-moderate depression was highest in Bangladesh as more than two-fifth participants reported being depressed during past 30 days compared to about one-third in Nepal. India had the lowest prevalence of depression of any duration. Explanations for this variation is not within the scope of the present study, however the lowest prevalence of depression among Indians could partly be due their better living standard (In terms of HDI) compared to most other south Asian nations. Material standard of living were reported to be associated with poor physical and mental health outcomes [21].

Prevalence of adequate amount (five servings per day) of fruits and vegetable consumption were remarkably low in all three countries. Fruit consumption at five servings/day was highest in India flowed by Nepal and Bangladesh. This could be due the fact that fruit is used as an essential component in traditional festivals and rituals practised by the Hindu communities across India and Nepal. Surprisingly, vegetable consumption (five servings/day) was lowest in India. Despite being a largely vegetarian country and being among the highest F&V producing nation, F&V account for less than one-tenth of total caloric intake among Indians [24]. This is mainly due to higher dependence on cereal diets, and availability issues due to poor preservation and supply chain infrastructure. Low F&V consumption may also be due to the dietary transition and rapid urbanization these countries are experiencing [22]. Over the past two decades, South Asian food intake patterns and dietary composition have undergone remarkable changes marked by a shift from a traditional cereal- and vegetable-based and low-meat diet to a high animal-based and low fruits- and vegetable- based diet [22]. In Bangladesh, consumption of F&V is currently 20% below the recommended daily intake [25]. In a Nepal, different studies have reported that about 66 to 99.2% of the population were not consuming recommended level of F&V [26, 27].

Consistent with previous researches, F&V intake was associated with higher prevalence of SRD in our analysis. Though data are not available for south Asian countries, evidence from developed countries indicate a positive dose—response relationship between F&V consumption with the risk of depression [28–30]. Among Swiss adults, consuming five servings of F&V a day was associated with lower odds of being highly or moderately distressed than consuming less than that [28]. Cross-sectional studies in the US and Canada also reported positive association between high fruit and vegetable consumption and lower mental distress [29, 30]. A recent meta-analysis on ten studies indicated that F&V consumption was inversely associated with the risk of depression [31].

### Strengths and limitations

To our knowledge, this is the first study to focus on F&V consumption and depression in South Asian population. Sample size was reasonably good for all three countries and was representative of the population. Therefore, the findings serve equally usefully to both policy makers and public health and nutrition researchers. As both short-terms and long-term depression were included, the findings produced a clearer picture on the magnitude of depression. Using the standard cut-off of five servings a day provides an internationally comparable prevalence of F&V consumption in the population. Despite these contributions, there are some important limitations that need to be considered to interpret the findings. As the survey was cross-sectional in nature, it does not allow making any causal inference of the associations. Also, since the direction of the association is not possible to know, low intake of F&V among subjects with depression could also be a result of reduced appetite. Last but not least, information on F&V intake and depression were self-reported, hence there remains a possibility of under/over reporting and recall error.

## Conclusion

In conclusion, prevalence of depression was high in all countries and was more prevalent among subjects who reported less than adequate level of F&V intake. An alarmingly large proportion of sample population did not adhere to the recommended amount of F&V consumption. Although the basic therapeutic approach for depression is pharmacological treatment, many clinical psychiatrists consider non-pharmacological approaches as an essential component of treatment. Non-pharmacological interventions such as dietary modification by encouraging higher consumption of F&V should be given more programmatic attention. The widespread production of F&V offers the opportunity for mass intervention of depression. In order to promote F&V consumption at national level, nutrition education and dietary behaviour changing programs can be integrated with community health projects. Addressing the barriers to access to F&V should also be taken into consideration in national food and nutrition security agenda. More in-depth studies are required to understand the barriers to and behavioral factors associated with F&V consumption.

## Abbreviations

DALYs: Disability-adjusted life years
GBD: Global burden of disease
LMICs: Low and middle-income counties
MDD: Major depressive disorder
NCDs: Non-communicable chronic disease
SRD: Self-reported depression
SWB: Social well-being
YLDs: Years lived with disability

## References

[1] Ferrari AJ, Somerville AJ, Baxter AJ (2013). Global variation in the prevalence and incidence of major depressive disorder: a systematic review of the epidemiological literature. Psychol Med.

[2] Moussavi S, Chatterji S, Verdes E, Tandon A, Patel V, Ustun B (2007). Depression, chronic diseases, and decrements in health: results from the World Health Surveys. Lancet.

[3] Murray CJ, Vos T, Lozano R (2012). Disability-adjusted life years [DALYs] for 291 diseases and injuries in 21 regions, 1990–2010: a systematic analysis for the global burden of disease study 2010. Lancet.

[4] Kastrup MC, Ramos AB (2007). Global mental health. Dan Med Bull.

[5] Ustün TB, Ayuso-Mateos JL, Chatterji S, Mathers C, Murray CJ (2004). Global burden of depressive disorders in the year 2000. Br J Psychiatry.

[6] Kessler RC, Aguilar-Gaxiola S, Alonso J, Chatterji S, Lee S, Ormel J, Ustün TB, Wang PS (2009). The global burden of mental disorders: an update from the WHO World Mental Health (WMH) surveys. Epidemiol Psichiatr Soc.

[7] Hidaka BH (2012). Depression as a disease of modernity: explanations for increasing prevalence. J Affect Disord.

[8] Bhugra D, Mastrogianni A (2004). Globalisation and mental disorders. Overview with relation to depression. Br J Psychiatry.

[9] Serrano Ripoll MJ, Oliván-Blázquez B, Vicens-Pons E (2015). Lifestyle change recommendations in major depression: Do they work?. J Affect Disord.

[10] Rubin RR, Wadden TA, Bahnson JL (2014). Impact of intensive lifestyle intervention on depression and health-related quality of life in type 2 diabetes: the Look AHEAD Trial. Diabetes Care.

[11] Akbaraly TN, Brunner EJ, Ferrie JE, Marmot MG, Kivimaki M, Singh-Manoux A (2009). Dietary pattern and depressive symptoms in middle age. Brit J Psychiatry.

[12] Mihrshahi S, Dobson AJ, Mishra GD (2015). Fruit and vegetable consumption and prevalence and incidence of depressive symptoms in mid-age women: results from the Australian longitudinal study on women’s health. Eur J Clin Nutr.

[13] Reddy MS (2010). Depression: the disorder and the burden. Indian J Psychol Med.

[14] Miller HE, Rigelhof F, Marquart L, Prakash A, Kanter MJ (2000). Antioxidant content of whole grain breakfast cereals, fruits and vegetables. Am Coll Nutr.

[15] Kodydková J, Vávrová L, Zeman M, Jirák R, Macásek J, Stanková B, Tvrzická E, Zák A (2009). Antioxidative enzymes and increased oxidative stress in depressive women. Clin Biochem.

[16] Maes M, Mihaylova I, Kubera M, Uytterhoeven M, Vrydags N, Bosmans E (2009). Increased 8-hydroxy-deoxyguanosine, a marker of oxidative damage to DNA, in major depression and myalgic encephalomyelitis/chronic fatigue syndrome. Neuro Endocrinol Lett.

[17] Ford DE, Erlinger TP (2004). Depression and C-reactive protein in US adults: data from the third national health and nutrition examination survey. Arch Intern Med.

[18] Ford ES, Mokdad AH, Giles WH, Brown DW (2003). The metabolic syndrome and antioxidant concentrations: findings from the third national health and nutrition examination survey. Diabetes.

[19] Uraiwan K, Nawi Ng, Hoang Van Minh et al. Fruit and vegetable consumption in rural adults population in INDEPTH HDSS sites in Asia. Glob Health Action. 2009; 2: 10.3402/gha.v2i0.1988. 10.3402/gha.v2i0.1988 PMC2785138 20027259

[20] Hall JN, Moore S, Harper SB, Lynch JW (2009). Global variability in fruit and vegetable consumption. Am J Prev Med.

[21] Vishwa M. Production high, but Indians eating less fruits and veggies. The Times of India. 2016. https://goo.gl/cljSll. Accessed 23 Apr 2016.

[22] Ghose B. Nutrition transition in South Asia: the emergence of non-communicable chronic diseases. Version 2. F1000Res. 2015; 4: 8. 10.12688/f1000research.5732.1 PMC4706051 26834976

[23] Sanni Y, Ghose B, Georges D, Vaibhav S, Michael E (2016). Trends and determinants of HIV/AIDS knowledge among women in Bangladesh. BMC Public Health.

[24] Weich S, Lewis G (1998). Material standard of living, social class, and the prevalence of the common mental disorders in Great Britain. J Epidemiol Community Health.

[25] Désirée van Gorp, Jeep Heida, Lisette Kuipéri-­‐Blüm et al. Business opportunitiesrelated to the developmentof retail and agro-food chains in Bangladesh. 2013. https://goo.gl/stgNsn. Accessed 23 Apr 2016.

[26] World Health Organization (2003). Research report on NCD risk factors surveillance in Nepal.

[27] Mishra SR, Neupane D, Bhandari PM (2015). Burgeoning burden of non-communicable diseases in Nepal: a scoping review. Glob Health.

[28] Aline R, Sabine R, Caroline LV, Meichun M, Monika E (2015). Associations between fruit and vegetable consumption and psychological distress: results from a population-based study. BMC Psychiatry.

[29] McMartin SE, Jacka FN, Colman I (2013). The association between fruit and vegetable consumption and mental health disorders: evidence from five waves of a national survey of Canadians. Prev Med.

[30] Rohrer JE, Stroebel RJ (2009). Does moderate fruit and vegetable intake protect against frequent mental distress in adult primary care patients?. J Altern Complement Med.

[31] Liu X, Yan Y, Li F, Zhang D (2016). Fruit and vegetable consumption and the risk of depression: a meta-analysis. Nutrition.

